# Hypertension in adult Fabry’s disease: is cardiotrophin-1 a diagnostic biomarker?

**DOI:** 10.1186/s12979-014-0027-3

**Published:** 2014-12-20

**Authors:** Monica Gioia Marazzi, Emanuela Galliera, Elena Vianello, Elena Dozio, Andrea Stella, Guido Tettamanti, Lorenza Tacchini, Massimiliano M Corsi Romanelli

**Affiliations:** Department of Biomedical Sciences for Health, Università degli Studi di Milano, Milan, Italy; Department of Biomedical, Surgical and Oral Sciences, Università degli Studi di Milano, Milan, Italy; IRCCS Galeazzi Orthopaedic Institute, Milan, Italy; Department of Sciences for Health, Università degli Studi di Milano - Bicocca, Milan, Italy; IRCCS Policlinico San Donato, San Donato, Milano, Italy

**Keywords:** Fabry disease (FD), Hypertension, Cardiotrophin-1 (CT-1)

## Abstract

**Background:**

Cardiotrophin-1 (CT-1), a cytokine produced by cardiomyocytes and non-cardiomyocytes in conditions of stress, can be used as a biomarker of left ventricular hypertrophy and dysfunction in hypertensive patients. Hypertension is one of the main adverse events in the third and last phase of Fabry’s disease (FD). We measured CT-1 in order to examine its correlation with the vascular and cardiac alterations at different ages and assess its potential for use as a biomarker of hypertension in FD.

**Findings:**

The level of CT-1 was clearly higher in hypertensive adults than in adult FD patients. FD patients show a small, non-significant decrease in plasma CT-1 with age, while in hypertensive patients CT-1 in plasma rises strongly and highly significantly with age.

**Conclusions:**

CT-1 can be considered a good biomarker of the progression of hypertension with age, but particular care is needed when following hypertension in FD patients, since CT-1 does not correlate the same way with this disease.

## Findings

### Introduction

Cardiotrophin-1 (CT-1) is a member of the interleukin-6 superfamily and activates gp130-dependent signaling, stimulating the (JACK/STAT) pathway and cardiac hypertrophic myocytes [1]. In conditions of stress, CT-1 activates different pathways in cardiac hypertrophic myocytes, leading to myocardial fibrosis, and contributing to the pathogenesis of hypertensive heart disease [2].

A recent study indicated that circulating CT-1 correlates with cardiac hypertrophy and vascular damage in hypertensive patients so it could serve as a biomarker of left ventricular hypertrophy and dysfunction in these cases [2,3]. CT-1 could therefore offer a new clinical and diagnostic approach for monitoring hypertension and its pathological effects [3-7].

Hypertension is one of the main adverse events occurring in the last phase of Fabry’s disease (FD). FD is a rare X-linked hereditary lysosomal storage disorder due to deficiency of α-galactosidase A (α-Gal A), resulting in the accumulation of globotriaosylceramide (Gb3), which leads to an inflammatory response [8] leading to a variety of clinical manifestations, ranging from cerebrovascular diseases to renal injury and cardiomyopathy [9-17].

FD nephropathy progresses with the severity of the disease, eventually resulting in chronic kidney disease, leading to hypertension. Usually untreated patients show three clinical phases of FD nephropathy, according to age [17]. In the first phase (childhood and adolescence) there is glomerular hyperfiltration; in the second (adults) there is renal involvement with proteinuria and lipiduria, and in the third phase severe renal and cardiovascular complications arise, leading to hypertension [14].

The vascular aspect of FD has been described [18,19] but there is still no ‘gold standard’ for monitoring the complications of hypertension. We therefore measured CT-1 in people with and without FD, developing hypertension with age, in order to examine the correlation between this cytokine and the involvement of the vascular and cardiac system at different ages and assess the potential for using it as a biomarker of hypertension in FD. CT-1 was positively associated with age in hypertensive patients, while in FD patients plasma levels took the opposite direction. The findings do indicate that CT-1 could be a good biomarker to monitor the progression of hypertension with age, but particular care is needed in FD patients because its levels do not correlate the same way with this disease.

## Patients and methods

### Patients

The population study comprised 18 FD (10 male and 8 female) and 34 (20 male and 14 female) not-FD hypertensive people, divided into two groups according to age (young, 3-30 years; adults, 40-65 years) and gender (young FD: 4 male and 4 female, young hypertensive: 8 male and 7 female, adult FD: 6 male and 4 female, adult hypertensive 12 male and 11 female. All FD patients had a confirmed diagnosis based on enzyme analysis and genotyping, and presented borderline hypertension at the time of the study. The hypertension population was defined as having systolic blood pressure (BP) ≥140 mm Hg and/or diastolic BP ≥90 mm Hg in three consecutive measurements. Fabry subjects present little if none ventricular hyperthopy, with Left ventricular wall below the pathological threshold (<12 mm).

### CT-1 assay

Plasma EDTA was obtained from all the participants. After centrifugation at 1500 g for 15 min, samples were rapidly frozen and stored at -20°C until assay. Plasma CT-1 was determined by enzyme-linked immunosorbent assay (ELISA) (BioVendor Research and Diagnostic Products, Brno, Czech Republic).

### Statistical analysis

The results are presented as mean ± standard deviation (SD). For between-groups comparisons we used Student’s two-tailed *t* test, taking p <0.05 as significant. Analyses were done with GraphPad Software (San Diego, CA).

## Results

**Figure 1:**
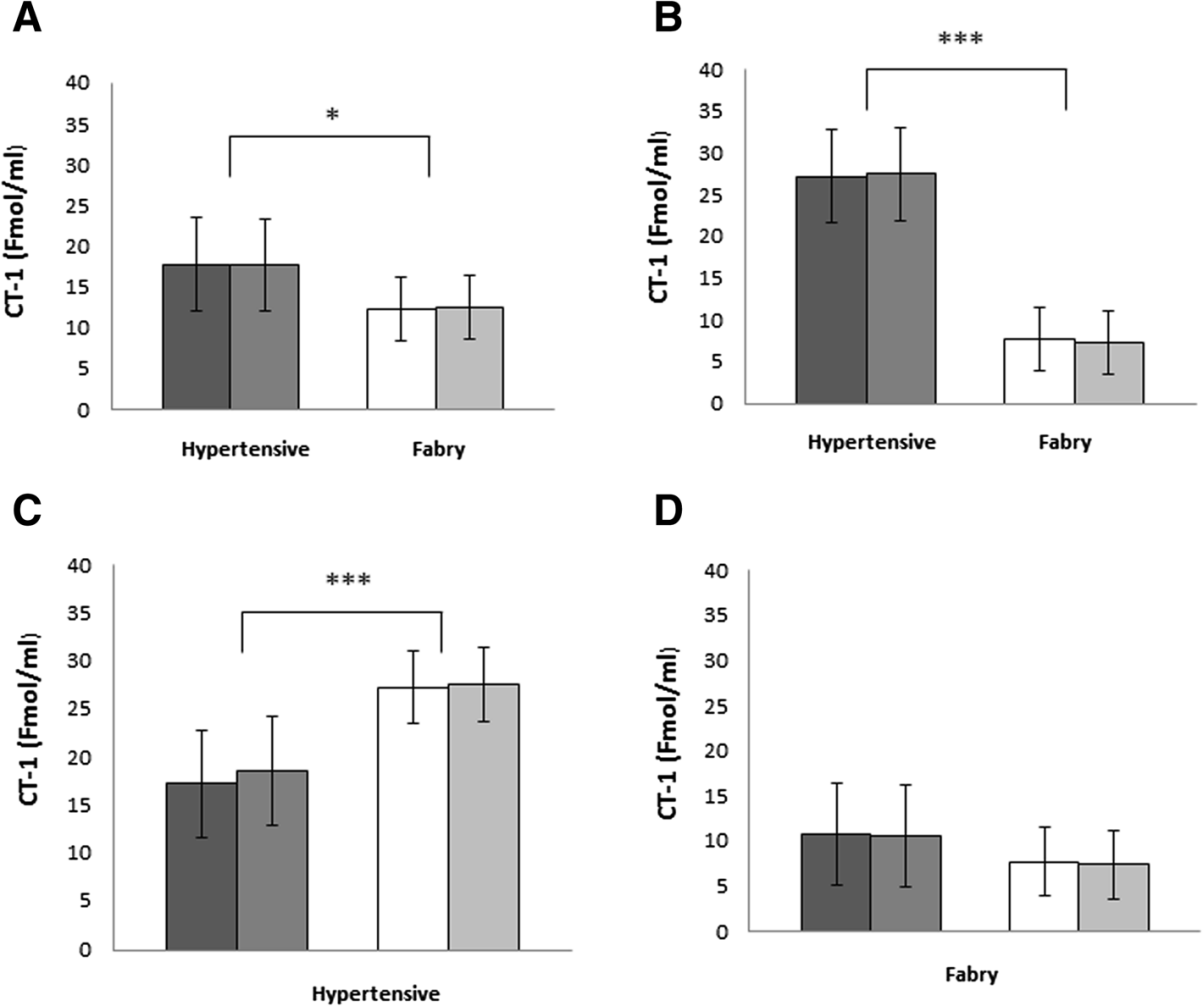
**Cardiotrophin-1 plasma levels in Fabry disease (FD) and hypertensive patients matched for age and sex.** Panel **A**: Cardiotrophin-1 (CT-1) plasma levels in young male and female (3-20 years old) hypertensive (black bar male/dark gray bar female) and FD patients (white bar male/pale gray bar female). Panel **B**: Cardiotrophin-1 (CT-1) plasma levels in adult male and female (30-65 years old) hypertensive (black bar male/dark gray bar female) and FD patients (white bar male/pale gray bar female). Panel **C**: Cardiotrophin-1 (CT-1) plasma levels in young male and female (3-20 years old, black bar male/dark gray bar female) and adult male and female (30-65 years old, white bar male/pale gray bar female) hypertensive patients. Panel **D**: Cardiotrophin-1 (CT-1) plasma levels in young male and female (3-20 years old, black bar male/dark gray bar female) and adult male and female (30-65 years old, white bar male/ pale gray bar female) FD patients.

CT-1 was measured in FD and hypertensive patients matched by age (Figure 1). Panel A depicts CT-1 plasma levels in young patients, which were slightly but significantly higher in those with hypertension (FD young 7.83 ± 4.47 Fmol/mL *vs.* hypertensive young 15.72 ± 7.93 Fmol/mL, p < 0.05). Panel B shows the CT-1 plasma levels in adults. CT-1 was clearly and highly significantly higher in hypertensive than FD adults (FD adults 6.14 ± 3.62 Fmol/mL *vs.* hypertensive adults 29.53 ± 3.92 Fmol/mL; p < 0.0001).

Figure 2 illustrates the CT-1 levels with age in the FD (panel A) and hypertensive patients (panel B). In FD patients plasma CT-1 decreased slightly but not significantly with age whereas in hypertensive patients the plasma levels showed a marked, highly significant increase with age (young *vs.* adults p < 0.0001). Data were divided and analyzed according also to the gender. No significative difference were observed between genders in any case.

## Discussion

The main finding of this study is that CT-1 is positively associated with age in hypertensive patients, while in FD patients plasma levels take the opposite direction.

In hypertensive patients there was an age-related tendency for CT-1 to rise, younger patients having lower levels than adults. This agrees with previous reports indicating CT-1 as involved in left ventricular hypertrophy and dysfunction in hypertensive patients [2,3,5] and could therefore be a good potential biomarker to monitor the development of hypertension. Since hypertension is a progressive condition that evolves with age, monitoring patients is very important for the clinical approach and treatment. In FD too there is a pathological progression with adult age, particularly in the cardiovascular system, leading to hypertension [11,14].

Our study is, to our knowledge, the first comparing circulating CT-1 level in FD and hypertensive patients, particularly in relation to age. While in non-FD hypertensive patients CT-1 plasma levels rose with age, FD patients showed the opposite pattern, young patients having higher CT-1 levels than adults. This agrees with a generally lower level of CT-1 in plasma of FD patients than hypertensive patients (as shown in Fig 1) . Thus, while CT-1 is considered a good biomarker in hypertensive patients for monitoring the cardiac dysfunction, FD patients develop hypertension differently, probably not involving the CT-1 mediated pathway. FD adults develop a variety of complications, being a consequence of progressive renal injury [20]. It is therefore possible that hypertension develops in FD following a different pathogenic mechanism from non-FD hypertensive patients, mainly related to cardiac dysfunction and, therefore, involving CT-1. CT-1 results were also divided and analyzed according to the gender in both FD and hypertensive patients, but no significative difference were observed between genders both in young and adult subject.

Our study has some limitations, including the small samples because FD is a rare disease, and the FD patients did not have severe cardiac complications. They also only had mild hypertension at the time, so we probably need patients with more serious hypertension to investigate the role of CT-1 further. For these reasons, our findings must be considered simply as preliminary observations that need thorough investigation to understand the potential for CT-1 as a hypertension biomarker in FD patients better.

In conclusion, CT-1 can probably be considered a good biomarker to monitor the progression of hypertension with age in association with left ventricular hypertrophy [21], but particular attention is needed when monitoring the development of hypertension in FD patients, where CT-1 does not correlate with this disease.

## Consent

Written inform consent was obtained from the patient for the publication of this report and any accompanying images.
